# India's Potential as a Leader in Cancer Care Progress in the Future: A Synthetic Interdisciplinary Perspective

**DOI:** 10.7759/cureus.70892

**Published:** 2024-10-05

**Authors:** Anshul Singh, Umesh Velu, Shirley Lewis, Roselin Nittala, Johnny Yang, Srinivasan Vijayakumar

**Affiliations:** 1 Radiotherapy and Oncology, Kasturba Medical College, Manipal Academy of Higher Education, Manipal, IND; 2 Radiation Oncology, University of Mississippi Medical Center, Jackson, USA; 3 Radiotherapy and Oncology, Cancer Care Advisors and Consultants LLC, Ridgeland, USA

**Keywords:** big data, genomic medicine, india, precision medicine, precision population medicine

## Abstract

This paper comprehensively analyzes India's potential to become a leader in cancer care in the Global South, particularly in precision population cancer medicine (PPCM). Through an interdisciplinary lens, it examines the current landscape of cancer care in India, highlighting its strengths, weaknesses, opportunities, and threats in this domain. This review explores the concept of knowledge translation and its importance in bridging the gap between knowledge generation and implementation in medical sciences and applies this to the Indian healthcare scenario. The review then delves into India's technological prowess, exemplified by its digital health initiatives such as the CoWIN (winning over COVID-19) app and the Ayushman Bharat Digital Mission, which provide a strong foundation for leveraging advanced technologies in healthcare. The authors discuss India's pharmaceutical industry, often referred to as the "pharmacy of the world," emphasizing its crucial role in global drug manufacturing and distribution. It also examines the country's emerging genomic research landscape, including initiatives such as GenomeIndia and the Indian Cancer Genome Atlas Foundation, which are pivotal for advancing personalized medicine. A significant portion of the review is dedicated to analyzing India's clinical trial ecosystem. It traces the evolution of regulatory frameworks governing clinical research in the country and highlights recent reforms that have made India an increasingly attractive destination for global studies, the potential adoption of innovative trial designs and artificial intelligence (AI)-driven analyses. Crucially, the authors confront the formidable obstacles inherent in India's complex healthcare landscape, illuminating the unique challenges that must be overcome. The review acknowledges India's underrepresentation in global clinical trials despite its large population and significant cancer burden. The issue of financial toxicity in cancer care is discussed, underscoring the need for affordable treatment options. The study also points out the nascent state of India's genomic databases, which account for only a small percentage of global genetic data. Despite these challenges, the authors posit that by effectively leveraging its information technology (IT) infrastructure, robust pharmaceutical sector, and large, diverse population, India has the potential to develop unique, country-specific solutions for cancer care. The study suggests that by fostering genomic research, strategically reforming its clinical trial ecosystem, and harnessing its digital capabilities, India could transform its cancer care landscape and emerge as a model for other developing nations in the Global South. In essence, this paper provides a roadmap for India's journey towards becoming a leader in PPCM, offering valuable insights for policymakers, healthcare professionals, and researchers in the field of oncology and precision medicine. Indeed, by using PPCM as a "pilot project," India can learn to use its new strategies to improve non-cancer care disease prevention, early detection, and improved and more cost-effective management. This approach could revolutionize cancer care in India and serve as a model for other developing nations in the Global South. By leveraging the strategies and technologies developed for PPCM, India could significantly enhance its healthcare system, highlighting the importance and urgency of improving cancer care in the region.

## Introduction and background

**Figure 1:**
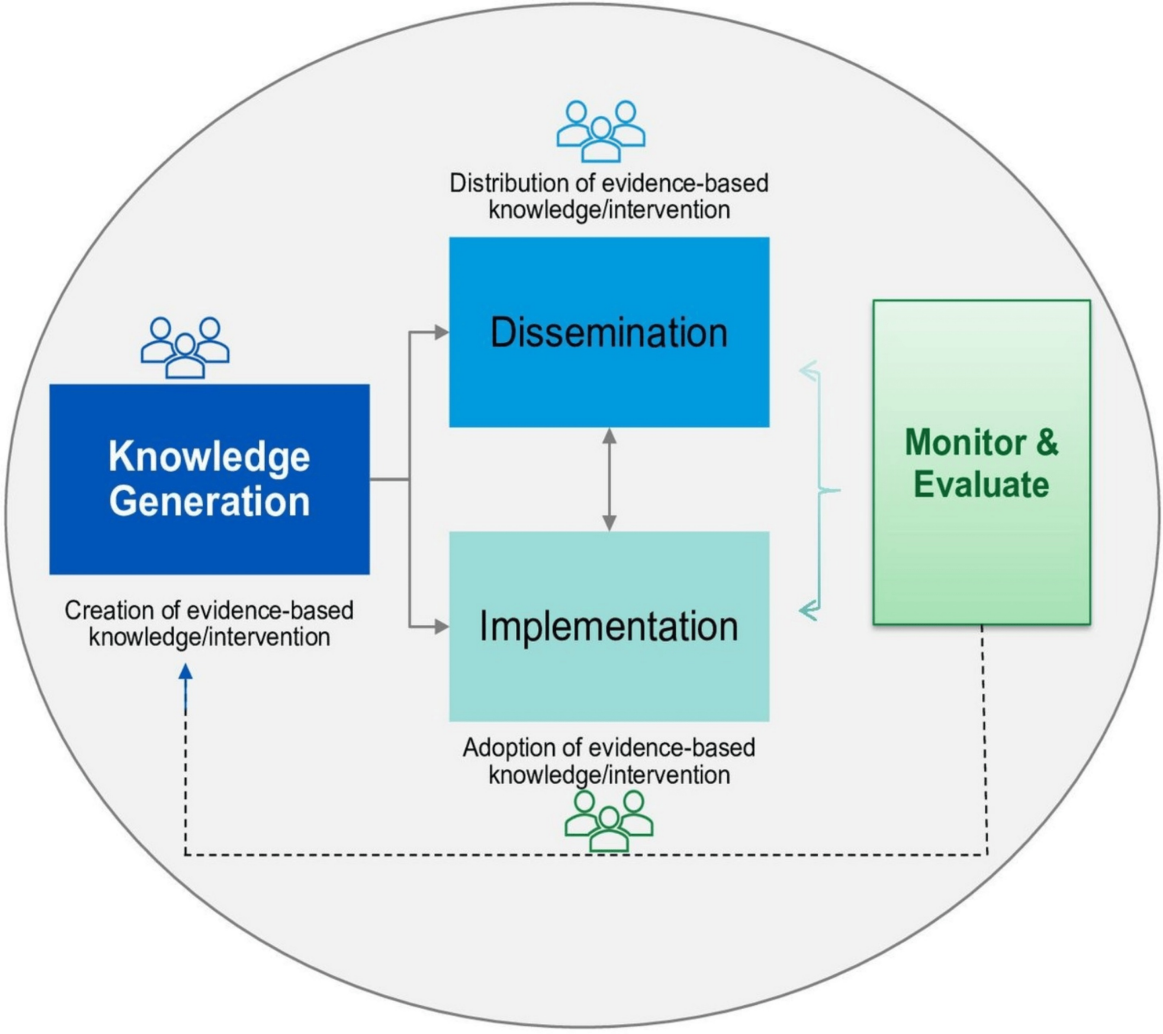
Integrated knowledge generation to disseminate–implement framework. The image has been reproduced from Daraz and Morshed [3] and is available via Creative Commons Attribution 4.0 International License.

The fast pace of scientific and technological innovations over the past 30 years has led to the potential birth of new ideas, innovations, thoughts, and even disciplines. This is especially true if one applies the "knowledge transfer" principles, sometimes referred to as "knowledge translation" (KT) [1]. It is a fact that there is an unacceptable delay and gap between "knowledge generation" (KG) and "knowledge implementation" (KI), coined the "knowledge-generation-implementation gap" (KGIG), especially in medical sciences [2]. Daraz and Morshed recently pointed out the need to streamline the definitions and strategies for KT. They identified three essential steps in this process: knowledge generation, knowledge dissemination, and knowledge implementation [3]. Figure 1 illustrates these concepts and how to decrease the KGIG.

**Figure 2:**
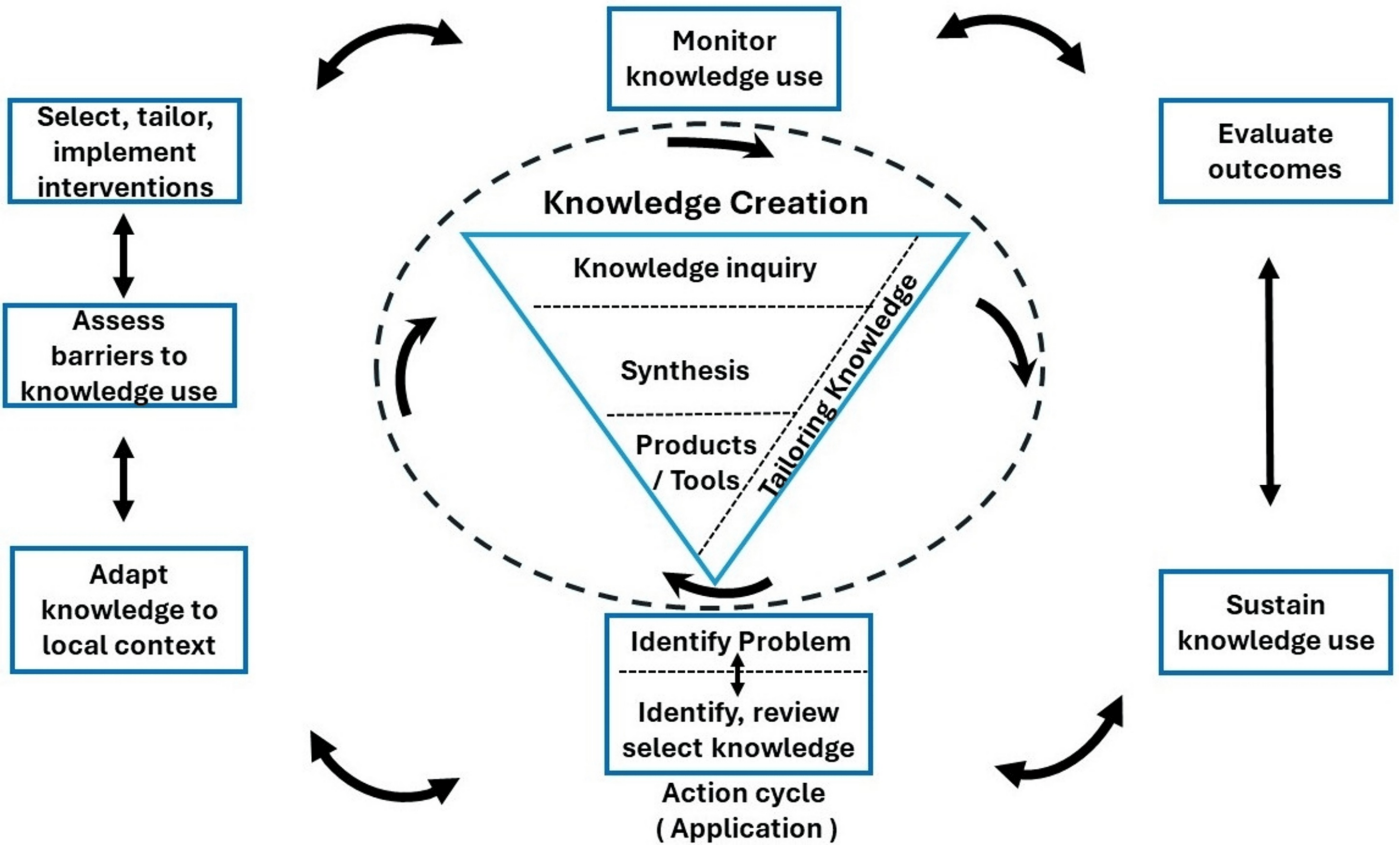
Knowledge to action process. The image has been modified from Graham et al. [4] and reproduced with permission from Kluwer. These contents were licensed under the CC-BY-SA License.

KG is not a simple and straightforward task, which is detailed in Figure 2 [3], and referred to as "knowledge creation" by Dammann [2] in their publication and illustrated by Graham et al. [4]. The many steps involved, from KG to ultimate KI and the steps to overcome KGIG, are detailed in Figure 2. Additionally, Table 1 lists the common phases of planned-action theories or models.

**Table 1:** The commonalities among planned-action theories. The tabulated data have been reproduced from Graham et al. [4] and reproduced with permission from Kluwer. These contents were licensed under the CC-BY-SA License.

The Common Phases of Planned-Action Theories
Identify a problem that needs addressing
Identify, review, and select the knowledge or research relevant to the problem (e.g., practice guidelines or research findings)
Adapt the identified knowledge or research to the local context
Assess barriers to using the knowledge
Select, tailor, and implement interventions to promote the use of knowledge (i.e., implement the change)
Monitor knowledge use
Evaluate the outcomes of using the knowledge
Sustain ongoing knowledge use

**Figure 3:**
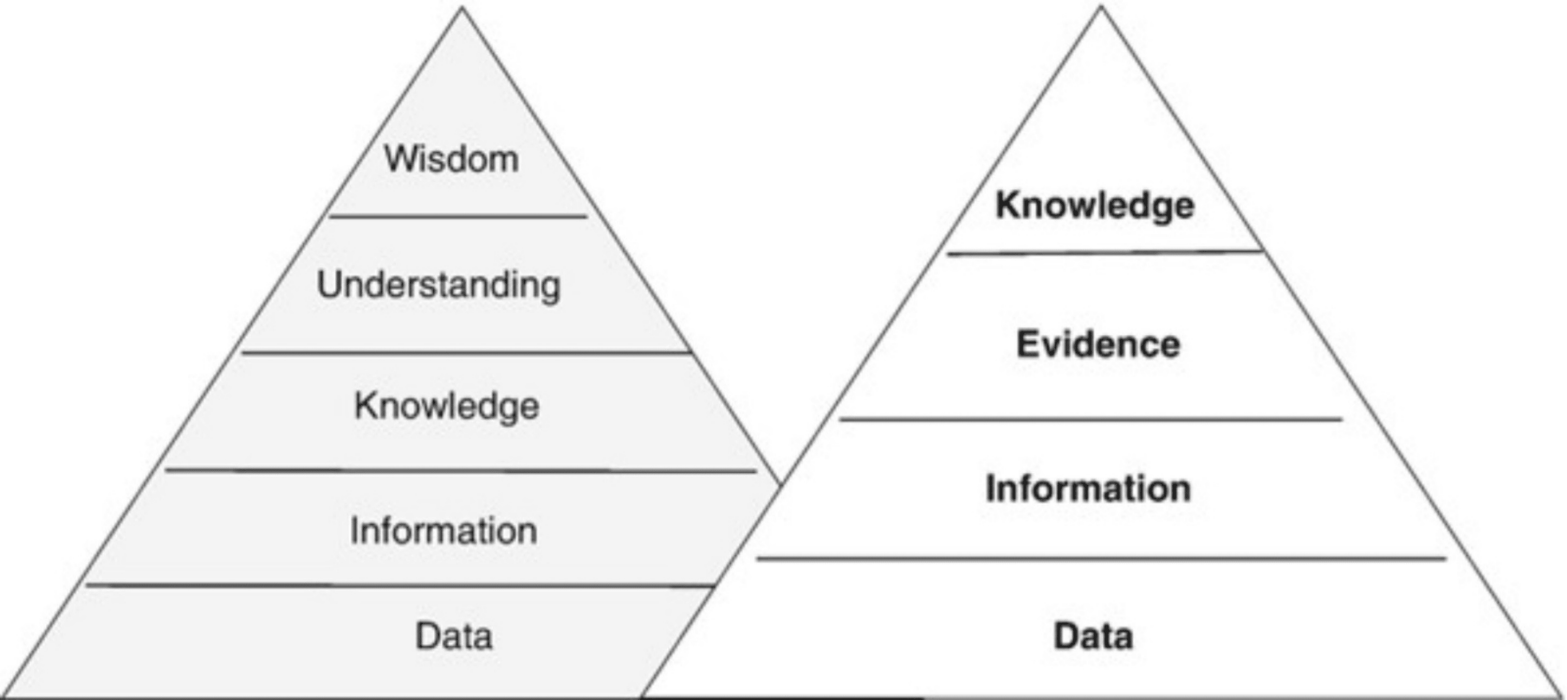
Ackoff's knowledge hierarchy (left) and the modification (right). The image has been reproduced from Dammann [2], and permission for the licensed content was obtained from the publisher, Springer Nature.

**Figure 4:**
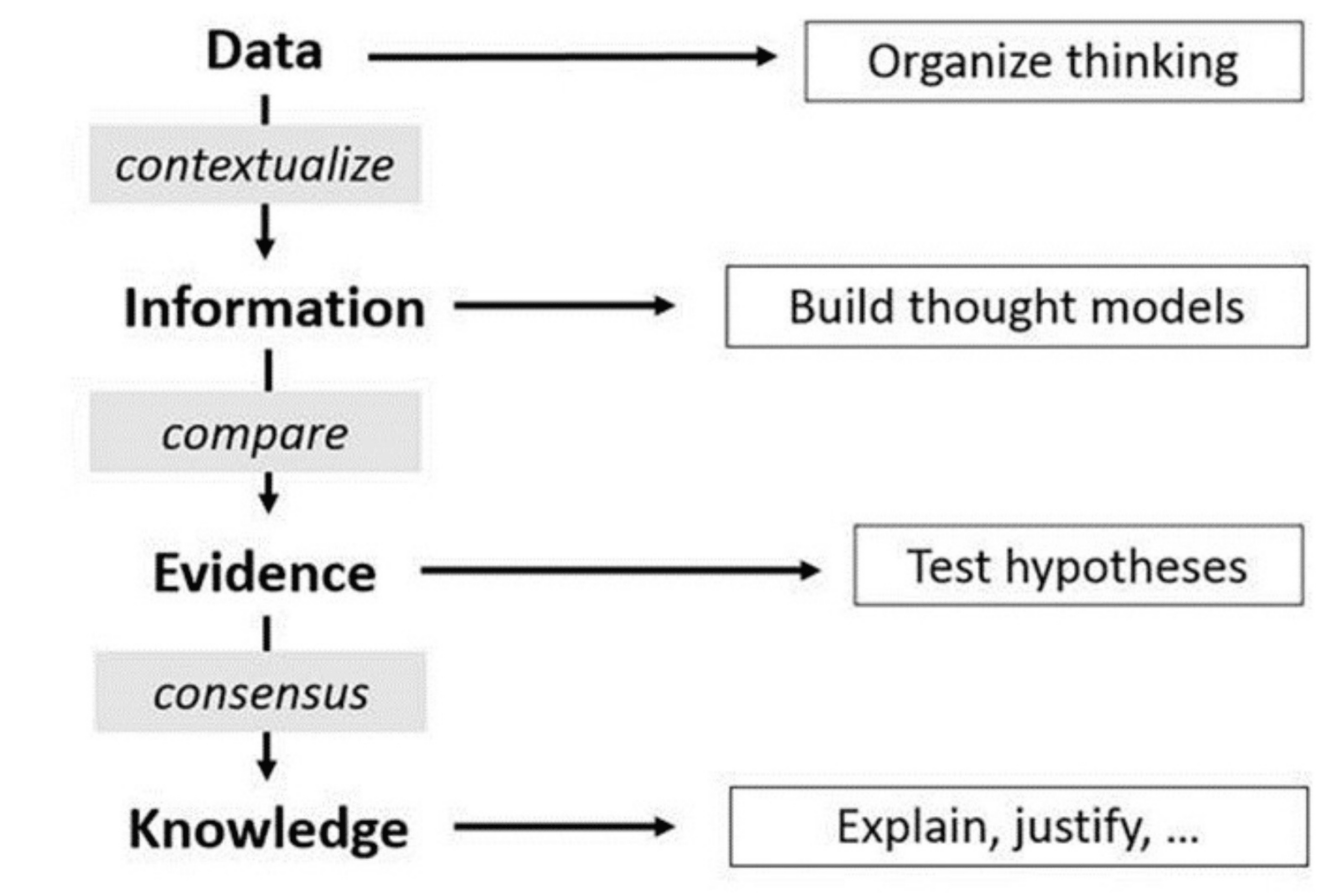
The framework for transitioning from data to knowledge (winged arrows) and what it is good for (straight arrows). The image has been reproduced from Dammann [2], and permission for the licensed content was obtained from the publisher, Springer Nature.

Knowledge inquiry has many more sub-steps. In the current era of information technology explosion, one must clearly distinguish among data, information, evidence, knowledge, and application (of that knowledge) [3,5]. The details of these ideas espoused by Ackoff and modified by Dammann are further detailed in Figures 3, 4 and Table 2. A fact often overlooked in medical sciences is the importance of "interdisciplinary research" (IR) [6-10]. In the context of cancer care in India, IR refers to the collaboration between different scientific disciplines to address complex healthcare challenges.

**Table 2:** Characteristics of data, information, evidence, and knowledge. The tabulated data have been reproduced from Dammann [2], and permission for the licensed content was obtained from the publisher, Springer Nature.

Concept	What Is It?	How Was It Produced?	By Whom?	Goal
Data	Numbers, symbols, text, images, sound recordings, unit values	Collected from field research, databases, measurements in experiments, from individuals, populations	Data collector	Use as raw data or for information generation, storage, curation, retrieval
Information	Data in context	Contextualization by making data useful and using them for specific tasks	Informatician, informaticist, statistician	Use as a source for answering questions, storage, curation, retrieval
Evidence	Useful, contextualized information	Comparison with standards, reference values, reference information	Scientist, theoretician, philosopher, interventionist, policymaker	Use for analysis and hypothesis-testing to support claims/hypotheses and decision-making
Knowledge	Evidence-based (predictive, testable, consistently successful) belief	Consensus based on reasoning and discussion		Justification

In this paper, we searched for and synthesized information, data, and available knowledge from different sources and disciplines. Effectively, we perform a KG that leads us to believe that cancer care in India can be improved at a relatively rapid pace over approximately 10-15 years. Furthermore, we hypothesize that using stepwise and continuous evaluative and re-finetuning processes [6], as detailed in Table 3, India can be one of the frontline leaders in cancer care outcome improvements. Among all the nations in the Global South, the necessary steps can be more easily planned, tested, and implemented in India than in any other country. This hypothesis is based on many fortuitous circumstances that have happened in recent times in India, and if properly taken advantage of, India can be a leader in innovating precision population cancer medicine (PPCM) that will go beyond mere disease management. This paper is an excellent example of an IR. India can thus lead the way in improving cancer care for the Global South. The benefits of an IR are expanded further in Table 3.

**Table 3:** Potential benefits from interdisciplinary research. The tabulated data have been reproduced from Dalton et al. [6] and are available under a Creative Commons Attribution 4.0 International License.

Benefit	Selected Studies	Summary
Problem-feeding	Grantham [11], Thorén & Persson [12]	Disciplines may rely on each other to generate hypotheses and solutions, especially when using shared heuristics.
Innovation and creativity	Collin [13], Jones [14], Nissani [15], Siedlok & Hibbert [16]	It is often maintained that interdisciplinarity leads to creativity and innovation. However, from the author's reading of the literature, it is unclear at this point if this increased creativity is based on a policy assumption, as noted by Siedlok & Hibbert, or borne out by the facts.
Disciplinary synthesis and integration	Winskel [17]	Disciplinary synthesis is often described as an ideal outcome of interdisciplinary research.
Enables a holistic approach suitable to societal-scaled problems	Collin [13], Nissani [15], Siedlok & Hibbert [16]	Interdisciplinary research teams have the potential to tackle complex societal-scale problems that monodisciplinary researchers are unable to. This, as discussed in this section, stems from both increasing specialization and increased complexity of the problems being investigated.
Social relations, networking, and flexibility	Siedlok & Hibbert [16]	Researchers may find great value in the social contact and interaction associated with working in teams with colleagues, as opposed to working in relative isolation. Interdisciplinary researchers may also appreciate the enhanced flexibility and freedom such collaborations offer.

**Figure 5:**
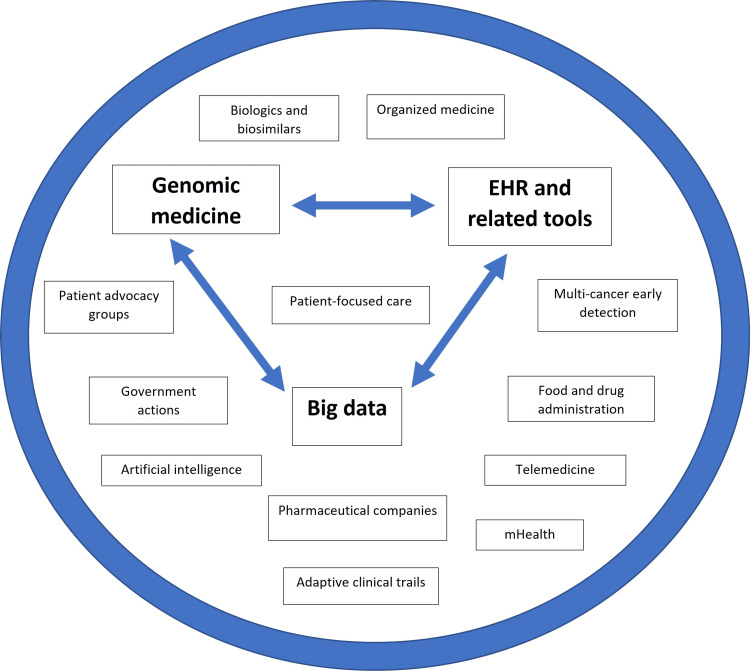
Components of precision population cancer medicine. The image has been reproduced from Yang et al. [19] and is available via Creative Commons Attribution 4.0 International License. EHR: electronic health records; mHealth: mobile health.

**Figure 6:**
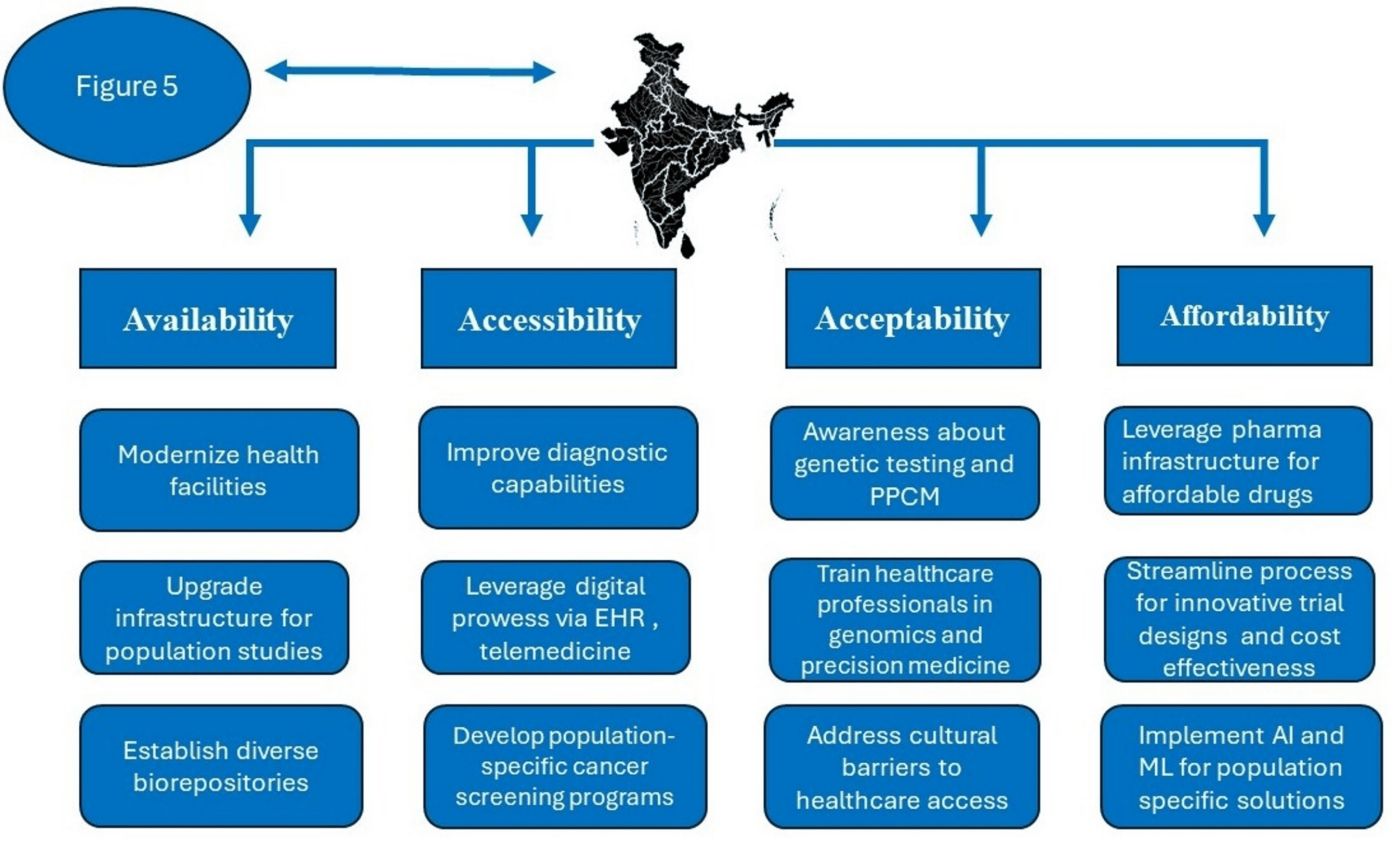
Key considerations for the success of PPCM in the Indian setting. This figure is an original work of the authors. EHR: electronic health records; PPCM: precision population cancer medicine; AI: artificial intelligence; ML: machine learning.

**Figure 7:**
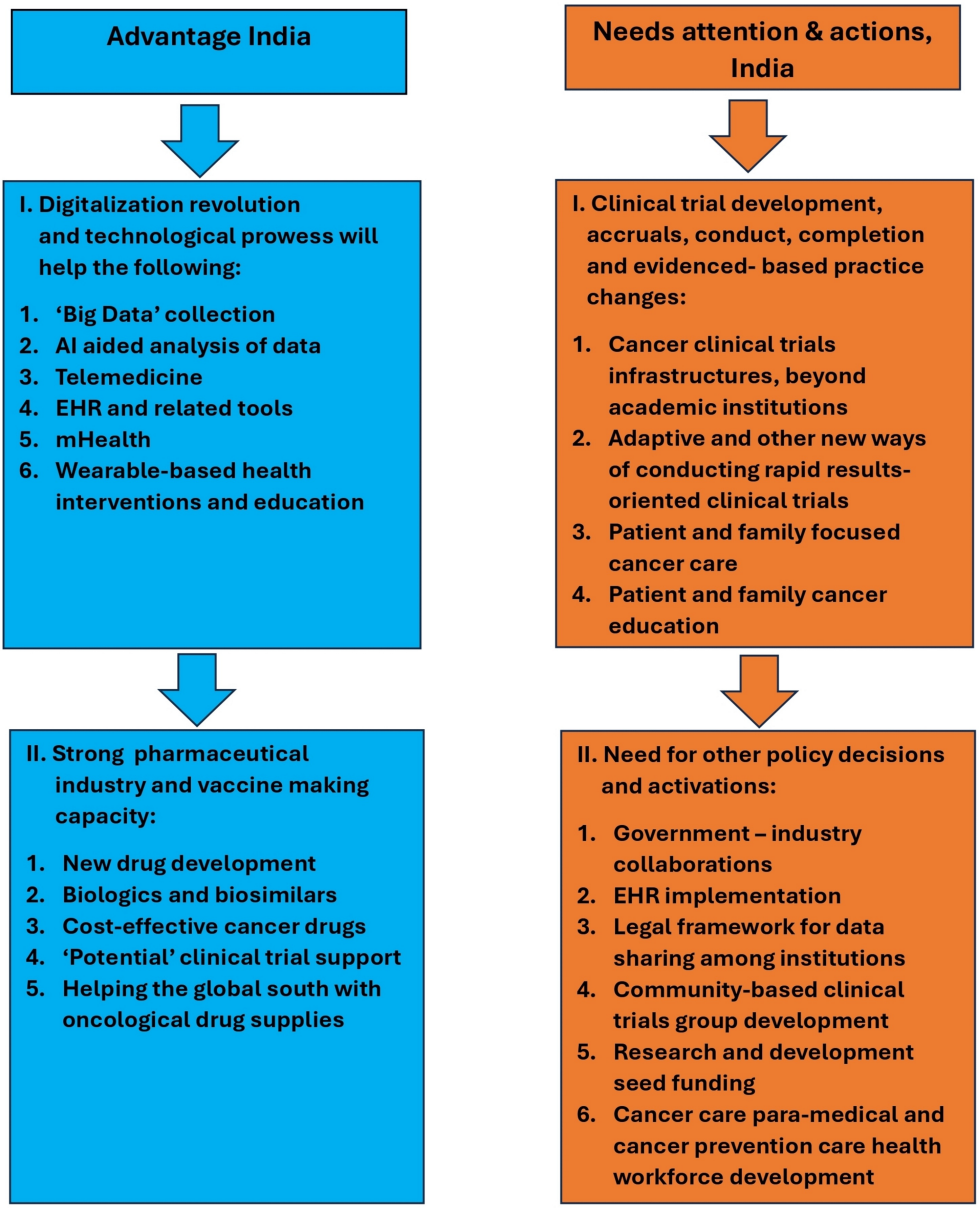
A visual representation of the challenges and the opportunities in India. This figure is an original work of the authors. AI: artificial intelligence; EHR: electronic health records; mHealth: mobile health.

Our hypothesis was formulated based on the information presented visually in Figures 5, 6 and the process outlined in the flowchart of Figure 7 [18,19]. Each item in the flowchart will be expanded in the subsequent sections. Figure 5 depicts the various components of precision cancer medicine (PCM), and Figure 6 illustrates the key considerations for success in the Indian and Global South settings. We emphasized the importance of understanding that PCM is more than genomic medicine (GM). With the innovations made in the last decade, the PCM's definition had to be changed, and that understanding and acceptance naturally led to an understanding of PPCM [18,19].

PPCM is a complex field with many components, detailed in Figure 5. Incorporating each one of these components and continuously updating those efforts as they further progress and evolve is crucial for making progress in cancer care. With its digitalization and IT prowess, India has promising potential in various areas of PCM. The key to harnessing this potential lies in swift and appropriate policy decisions coupled with rapid implementation. Tailoring these approaches to thrive in the Indian setting is represented in Figure 6. The transformative role of digital health initiatives in cancer care is inspiring, offering a hopeful outlook for the future of cancer care in India.

The objectives of our synthetic perspective here are (a) to draw attention to the potential opportunities to improve cancer care in India, (b) to highlight the steps that need to be taken to achieve those opportunistic goals, (c) to use those experiences to apply to the Global South with required local nuanced modifications, and finally (d) to fortuitously utilize these experiences to improve cancer care for the disadvantaged populations that exist within advanced societies.

## Review

Materials and methods

A comprehensive literature search was conducted using MEDLINE/PubMed and Google Scholar databases. The search strategy employed various combinations of keywords, including "precision medicine," "personalized medicine," "genomic medicine," "targeted therapy," "cancer," "oncology," "neoplasm," and "India." Additional terms such as "biomarkers," "molecular diagnostics," "genomic profiling," and "pharmacogenomics" were also utilized. Boolean operators (AND, OR) were used to refine the search. Reference lists of relevant articles were manually screened to identify additional studies. Articles were selected based on their relevance to PCM in the Indian context, focusing on recent developments and challenges. Initial searches were done by the corresponding author in December 2023 prior to a presentation at an international conference and were repeated and updated between June and August 2024 by all authors.

Inclusion and exclusion criteria

The search was limited to English-language articles published between 2000 and 2024, with no other specific filters being used. Commentaries, letters to the editor, and unpublished reports were excluded.

India's COVID-19 vaccine leadership story as a lesson for the pharma leadership of India: digital prowess of India

**Figure 8:**
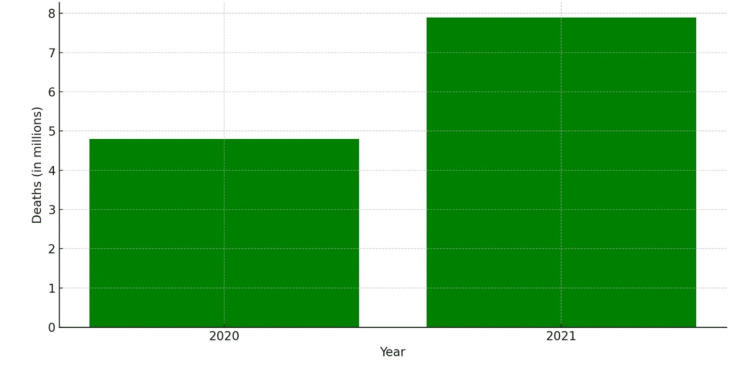
COVID-19 and other pandemic-related mortality. The data for the graph have been sourced from Mohsen et al. [20]. COVID-19: coronavirus disease 2019.

A recent publication in The Lancet [20] provided an overview of the COVID-19 aftermath. COVID-19 emerged as the second-leading cause of age-standardized death globally in 2021, with 94.0 deaths per 100,000 population. This shifted the rankings of leading causes, lowering stroke to third. The estimates show 4.80 million deaths due to COVID-19 globally in 2020 and 7.89 million in 2021. A net reduction of 1.6 years occurred in global life expectancy between 2019 and 2021, primarily due to increased death rates from COVID-19 and other pandemic-related mortality (Figure 8).

The Indian pharmaceutical industry played a pivotal role in the global fight against the virus by rapidly scaling up the production of vaccines. The industry's ability to mass-produce affordable and high-quality vaccines globally led to the then UN Secretary-General Antonio Guterres famously describing India's vaccine production capacity as the "best asset that the world has" [21]. The Indian pharmaceutical industry boasts an extensive network of over 3,000 companies and 10,500 production units. These firms contribute an impressive 20% of the world's generic drug supply and approximately 62% of the global vaccine output. Additionally, this robust infrastructure enables the sector to manufacture medications at remarkably lower costs than other major global markets. With over a century of existence, it has rightfully earned the appellation "the pharmacy of the world" [22,23].

Another essential asset in India's repertoire in the fight against the COVID-19 pandemic has been the CoWIN (winning over COVID-19) app, a digital platform acclaimed worldwide. The app supports registration, immunizations, appointments, and digital vaccine certificates. It has been instrumental in vaccinating over 960 million people. Its success owes a lot to the digital platform, the Electronic Vaccine Intelligence Network (eVIN), which digitized the vaccine supply-chain network using mobile technology. Thus, the pandemic unexpectedly accelerated the emphasis on building a robust digital health infrastructure (DHI) to address the emerging needs of a digitally connected globe [24-26].

The World Health Organization's global strategy on digital health 2020-2025 report has also acknowledged the importance of digital transformation and DHI's role [27]. Virtual medical care, smart wearables, and remote health monitoring, in conjunction with cutting-edge information and communications technologies, have already initiated a continuum of this bionic transformation. The healthcare sectors globally are realigning their strategies. They envision digital health solutions and innovations as unprecedented opportunities to remedy the age-old healthcare afflictions of availability, accessibility, acceptability, and affordability [28]. The BharatNet project and the development of the semiconductor industry positions India as a significant player in the worldwide digital ecosystem. Key advancements include implementing next-generation communication technologies and satellite networks, enhancing broadband connectivity and bridging the digital divide between rural and urban areas. The healthcare sector stands to capitalize the most on the immense potential of this digital inclusion [29-31]. The Government of India launched the Digital India initiative in 2015, which has created significant progress and transformation in the country's technological landscape. The National Health Policy 2017 envisioned a fully digitized healthcare system and set India's National Digital Health Mission in motion [31,32]. This subsequently evolved into the Ayushman Bharat Digital Mission (ABDM) [33], which includes digital components such as Health ID for storing and sharing health data, health facility registry for public and private healthcare providers, healthcare professionals registry, and health records accessible via a mobile application. The policy also emphasizes the need to automate, digitize, and connect diagnostic labs through the Laboratory Information Management Systems (LIMS) [34].

Additionally, to foster innovation and incubate the startup culture, a Digital Health Sandbox [35] initiative allows healthcare technologies and products to be tested in a controlled environment to assess consumer and market reactions before larger-scale deployment. This initiative is supported by the Health Data Management Policy that aims to establish a robust framework for the secure management of health data within the ABDM. ABDM seeks to establish a secure, interoperable, and comprehensive digital health ecosystem by 2025.

These initiatives collectively aim to enhance healthcare access, quality, and innovation through digital transformation, addressing the unique challenges of the Indian healthcare system [31-35]. The following section delves deeper into these unique issues, exploring their specific manifestations and implications within the Indian context.

When viewed through the lens of its unique socioeconomic fabric, India's vast and varied health sector presents a complex panorama that demands creative, locally crafted intervention. Over the past decade, oncologists have witnessed remarkable advancements in cancer drug development and approvals. However, these breakthroughs are associated with escalating costs and invariably widen inequities in access to cutting-edge therapies. This disparity in access to effective treatments has led to a troubling scenario where cancer outcomes vary dramatically across the globe [36].

In India, cancer care is particularly plagued by financial toxicity [37]. This economic burden diminishes patients' quality of life and strains household finances, forcing families to de-prioritize other essential needs and sometimes abandon treatment altogether [38]. If the patient happens to serve as the household's primary income, the implications of financial toxicity can be unbelievable. To address these challenges, innovative strategies are urgently needed. These may include pooled procurement, national-level negotiations, group contracting, and rigorous identification of high-quality generics and biosimilars. Strengthening regulatory processes to ensure consistent drug quality and supply, coupled with fair pricing initiatives, could significantly alleviate the issues identified in recent studies [39]. However, as the landscape of cancer therapy evolves, new challenges and opportunities arise.

While these strategies aim to address the financial toxicity of cancer care in India, they primarily focus on traditional treatments. One promising area that has emerged in recent years is immunotherapy, which leverages the patient's immune system to combat cancer [40]. Advances in genomics and sequencing have enabled the identification of cancer-specific neoantigens, which can be targeted by personalized immunotherapies such as vaccines and engineered T-cell therapies [41]. This integration of personalized oncology and immunology has opened new avenues for precision immunotherapy, including neoantigen-targeted vaccines and using mutational signatures as response predictors [42]. Immunogenomics, which uses genomic data to guide immunotherapy, has become central to precision cancer treatment, leading to accelerated approval of checkpoint inhibitors and improved patient selection for immunotherapy [43]. However, it is important to note that these cutting-edge therapies introduce new complexities regarding cost and accessibility. Their high cost presents a significant barrier to access for many patients and healthcare systems worldwide, particularly in resource-limited settings [44].

An innovative low-dose immunotherapy trial conducted in India has demonstrated the potential to increase the accessibility of these novel agents without compromising efficacy [45]. This recent study explored the use of nivolumab at just 6% of the FDA-approved dose for head and neck cancer patients, dramatically reducing treatment costs to less than 10% of standard monotherapy [46]. Through the ingenious vial-sharing approach, the trial ensured efficient resource utilization, making the drug both accessible and affordable under India's national public health insurance [47]. This pragmatic approach, born out of necessity in a resource-limited setting, not only showcases India's untapped potential in clinical trials but also underscores the value of India's varied demographics in PPCM research. This, in turn, aligns with our hypothesis of how India can lead the Global South towards more equitable cancer care.

Building on this innovative approach to clinical trials and leveraging its unique demographic advantages, India is well-positioned to extend its influence in the global cancer care landscape through its robust pharmaceutical sector. India's robust pharmaceutical industry, renowned for its manufacturing capabilities, is poised to play a pivotal role in this transformation. The country's growing expertise in biosimilars, which are essential components of PPCM, presents a substantial opportunity. Industry reports suggest that the global biosimilar market could offer a $240 billion opportunity for Indian biopharmaceutical companies, with the domestic market projected to reach $40 billion by 2030 [48]. India can lead the way in developing region-specific strategies [49] to ensure that life-saving cancer treatments become accessible to all who need them.

In the grand theater of global health, innovative, affordable biosimilars take center stage as one of the fundamental enablers of precision cancer care. They are the great equalizers, bridging the gap between cutting-edge science and universal access. As the curtain rises on this new era, India, armed with its pharmaceutical might and digital ingenuity, stands ready to lead the charge.

The need for India-specific cancer genomics data

The Human Genome Project (HGP), launched in 1990, was a pioneering global collaborative research project [50]. Its impact reverberates across the world, making a significant transformation in the approach to biomedical research and unraveling an extensive realm of biological knowledge. The initial step in advancing precision medicine is establishing a genomic database. Cancer, a genetic disease with the interplay of lifestyle and environmental factors, mandates population-specific genetic profiling. Moreover, to balance the regional, economic, and healthcare infrastructure disparity necessitates country-specific biobanks. These can help optimize healthcare delivery based on a given population's specific needs and challenges.

**Figure 9:**
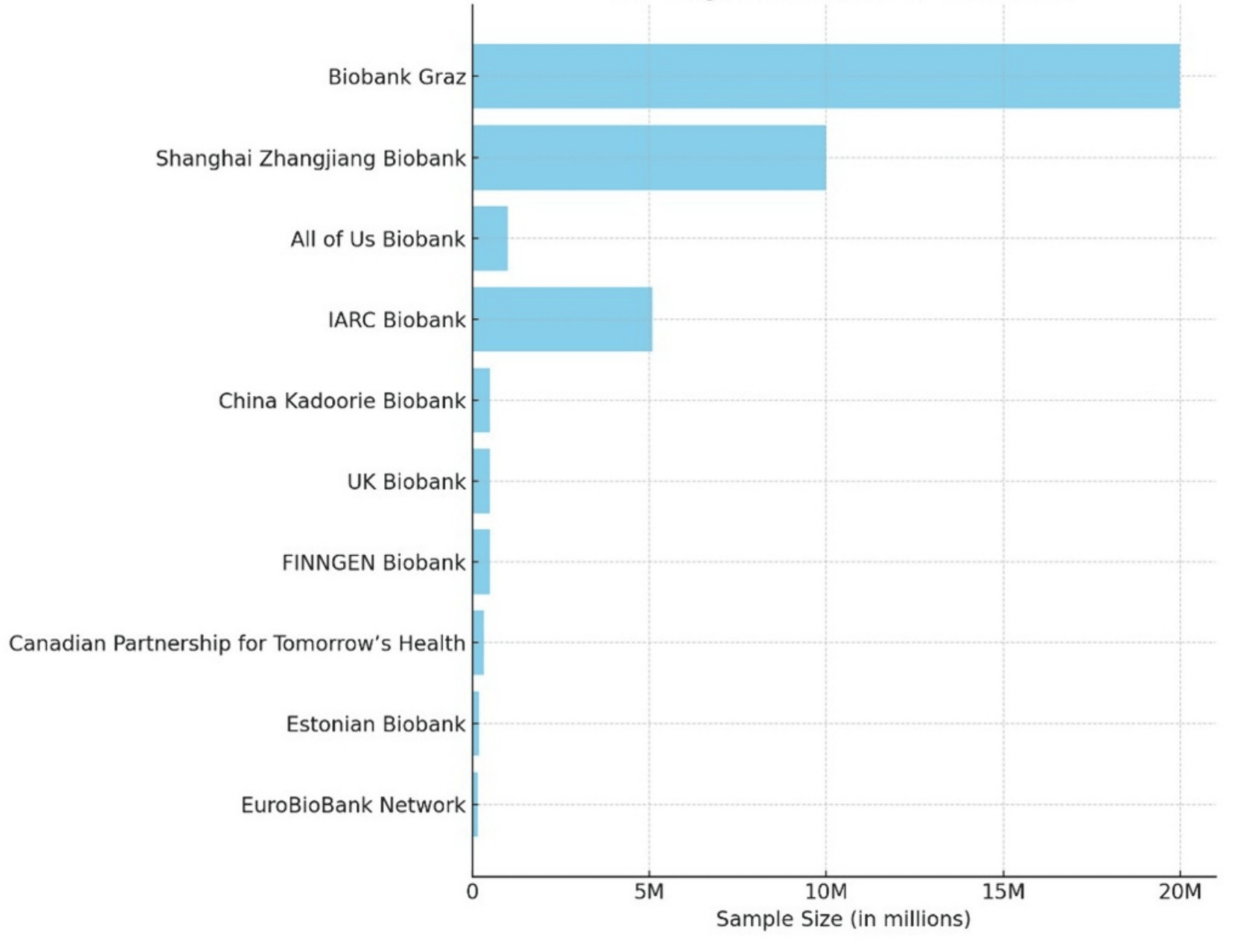
The 10 largest biobanks across the globe. The graph is adapted from the data available at biobanking.com [55]. IARC: International Agency for Research on Cancer; ​​UK Biobank: United Kingdom Biobank; FINNGEN: Finnish Genomic Research Organisation.

Additionally, each country's unique regulatory environment warrants careful navigation of ethical and legal norms. Region-specific initiatives such as the All of Us Research Program [51], the UK Biobank [52], the 1+ Million Genomes Initiative [53], and the Qatar Biobank [54] are essential advancements. Figure 9 depicts the 10 largest biobanks [55] and their relative "sizes," or rather, the volumes of tissue samples.

The Qatar Biobank stands as a beacon of innovation and foresight in medical research on prevalent health issues in Qatar. Under the guidance of experts from Imperial College London, a pilot study was launched in 2012. As a long-term, population-based study, it aims to collect high-quality biological samples and detailed data from 60,000 Qataris and long-term residents who have lived in Qatar for at least 15 years [56]. The strategic vision behind Qatar Biobank is laudable. Other nations can draw inspiration from how proactive investment and international collaborations in biobanking can yield long-term benefits for national and global health.

Dhup et al. [57] have echoed the same sentiment and highlighted that despite India's 1.3 billion population, it accounts for a mere 0.2% of global genetic databases. The report underscores the fact that with 1.5 million new cancer cases and 800,000 deaths each year, the specter of cancer looms large over India. By 2025, this immense burden will swell by 12.8% [57]. The moment is opportune for India to address the escalating cancer crisis, and the GenomeIndia project [58], Indigen (Genomics for Public Health) [59], and Indian Cancer Genome Atlas Foundation [60] are commendable initiatives. These genomic datasets could pave the way for groundbreaking targeted therapies and diagnostic tools tailored to the Indian population, offering a beacon of hope in the fight against cancer.

Components of precision cancer care

In a historic moment on January 30, 2015, President Obama unveiled a groundbreaking albeit audacious endeavor to usher in a new era of healthcare: The Precision Medicine Initiative [61]. The US National Cancer Institute defines precision medicine as "a form of medicine that uses information about a person's genes, proteins, and environment to prevent, diagnose, and treat disease" [62]. There are differing interpretations and definitions of the concept; the core ideology is to optimize treatment by considering the bespoke biological attributes of each patient [63].

Precision oncology, a subspecialty within the broader field of precision medicine, is making significant strides in delivering targeted, individualized cancer treatments [64]. The transformative potential of precision oncology is not just a promising concept but a reality. Zhang et al. [64] studied the trend of annual publications on precision oncology from 2012 to 2021. In 2012, a mere dozen papers were published, accounting for a paltry 0.17% of the total publications. Fast-forward to 2021, and the landscape had transformed, with an astonishing 1,522 papers, a staggering 126.75-fold increase! This buoyant trend of annual publications inspires and motivates researchers with the transformative potential of precision oncology in cancer care.

Cancers mirror the principles of Darwinism through a complex process of somatic evolution [65]. This underpins the crucial role of genomic and molecular profiling as foundational elements for detecting actionable aberrations [66].

**Figure 10:**
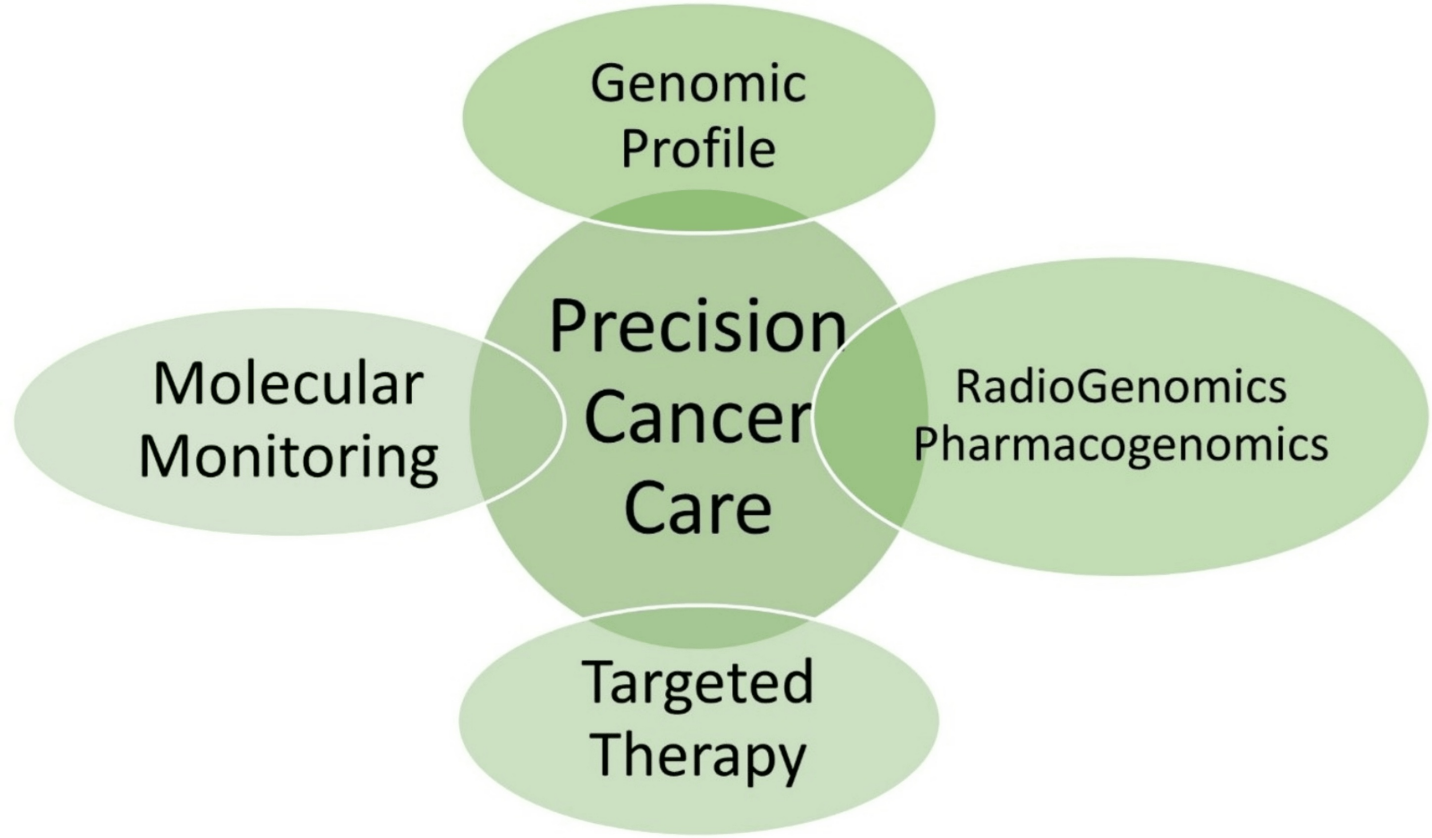
The cornerstones of precision medicine. The diagrammatic intersection of genomic profile, targeted therapy, radiogenomics, pharmacogenomics, and molecular monitoring in precision cancer care, adapted from Liu et al. [67] and Pirmohamed [68].

Enter radiogenomics and pharmacogenomics, the vigilant sentinel and the wise alchemist [67,68]. Radiogenomics studies the relationship between an individual's genetic makeup and their response to radiation therapy. Pharmacogenomics, conversely, is the study of how an individual's genetic makeup affects their response to drugs. When all of these come together, they provide a panoramic view of the tumor's landscape, enabling focused targeting of tumors with modern radiotherapy and immunotherapy. Furthermore, liquid biopsies coupled with big data and artificial intelligence-based analytics enable monitoring and fine-tuning the therapy as per individual patient response. These cornerstones of precision medicine genomic profiling, radiogenomics, pharmacogenomics, targeted therapy, and molecular tracking are heralding a new transformative era in cancer care, which is summarized in Figure 10.

Big data and precision medicine

The adage "data is the new oil" was created by British entrepreneur Clive Humby [69]. The cryptic similarities in their extraction, processing, and waste generation are only undone by the limitlessness of the former as opposed to the exhaustible nature of the latter. The term "big data" has various interpretations, partly due to the subjective nature of the word "big." Some definitions emphasize the quantitative aspect, concentrating on the sheer volume of data. Other definitions take a qualitative approach, focusing on the size or intricacy of data that surpasses the capacity of conventional data analysis techniques to process it effectively [70]. In the context of biomedical research, big data and AI are revolutionizing the way clinicians and researchers understand and treat diseases, particularly cancer, providing new insights and improving patient outcomes through clinical trials. This approach has limited scope when dealing with "omics" data, as it is of a higher dimension and is nonlinear in nature [71].

Analyzing these vast datasets helps identify patterns and correlations that would be impossible to detect with traditional methods. Advanced analytical tools and machine learning algorithms are employed to process and interpret these massive datasets, leading to new insights into disease mechanisms and treatment responses. Furthermore, it also aids drug development, designing adaptive clinical trials, and improving diagnostic accuracies and treatment outcomes.

Clinical trials and cancer care clinical trials in India: the past, present, and future

India's clinical trial journey is a captivating story of regulatory transformation and strategic reforms. It all began with the Drugs and Cosmetics Act (DCA) of 1940 and the Drugs and Cosmetics Rules of 1945 (DCR) [72], which focused on drug import, manufacture, distribution, and sale, leaving clinical research unregulated. Over the years, India has made significant strides in clinical trials, with the establishment of regulatory bodies and the implementation of guidelines to ensure trial safety and ethical conduct. Today, India is a key player in the global clinical research landscape, with a growing number of trials being conducted in the country.

A pivotal moment came in 1970 when India replaced its colonial patent laws with the Indian Patents Act [73], fostering a "process patenting regime." As a result, India became a hub for generic drugs. This warranted the introduction of Schedule Y in 1988 [74], mandating Phase III clinical trials for new drug approvals. In 2005, the government amended Schedule Y [75] to establish a robust regulatory framework, introducing a four-phase clinical trial scheme and formalizing the Central Drugs Standard Control Organisation-Good Clinical Practice guidelines (CDSCO-GCP) [75,76], thus laying the foundation for structured clinical research in India. Simultaneously, the Indian Council for Medical Research (ICMR), the apex body for biomedical research, issued the Guidelines for Biomedical and Health Research Involving Human Participants in 2000, later revised in 2006 to enhance ethical standards and research quality [77].

**Figure 11:**
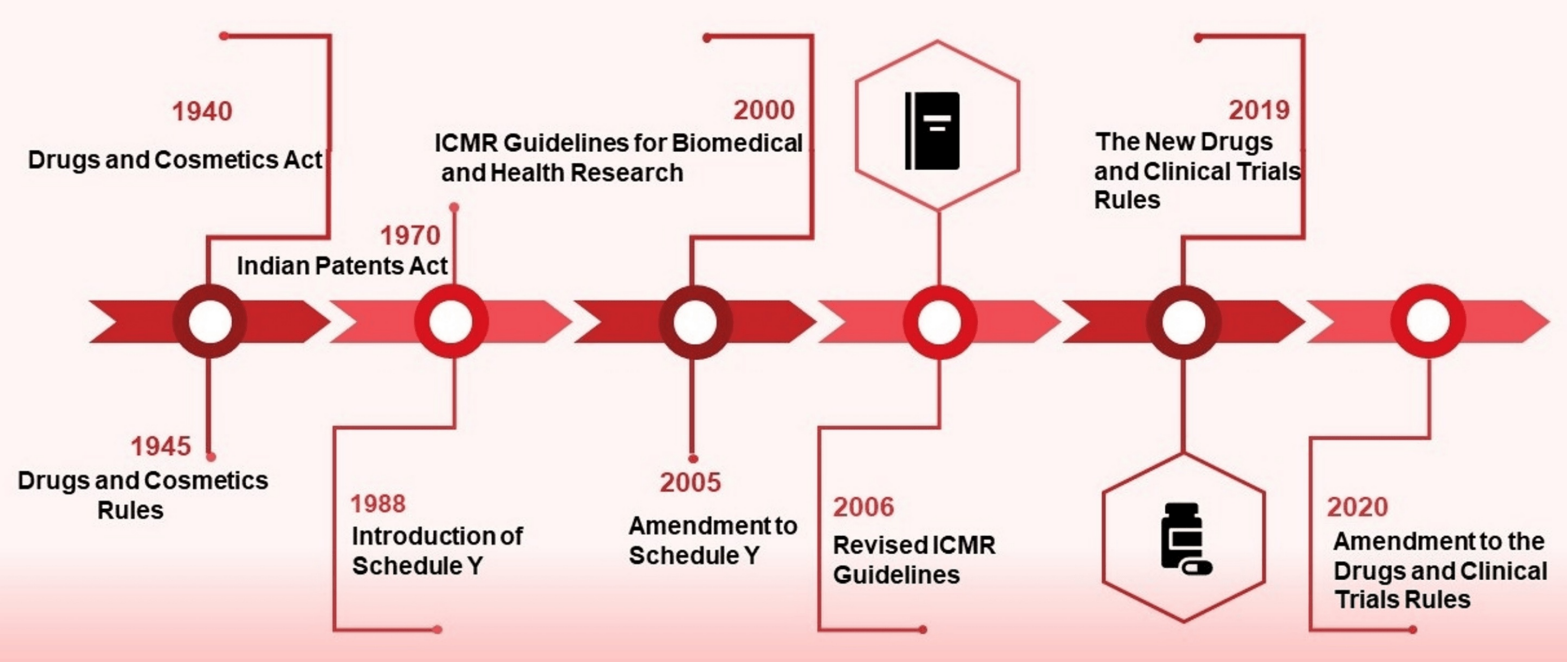
Timeline of the regulatory transformations in the Indian pharma sector. The graph is adapted from the data available from multiple prominent sources [72-79]. ICMR: Indian Council for Medical Research; Schedule Y: "Y" is simply the alphabetical designation for this particular schedule.

The most recent updates are the New Drugs and Clinical Trials Rules 2019 [78] and an amendment to the DCR in 2022 [79], which have reduced the duration of necessary regulatory approvals for bringing a new drug into the market to three to six months. Following the new regulations, the number of clinical trial sites has increased by 40% between 2014 and 2022 [80]. The timeline in Figure 11 gives a comprehensive overview of India's key regulatory transformations.

**Figure 12:**
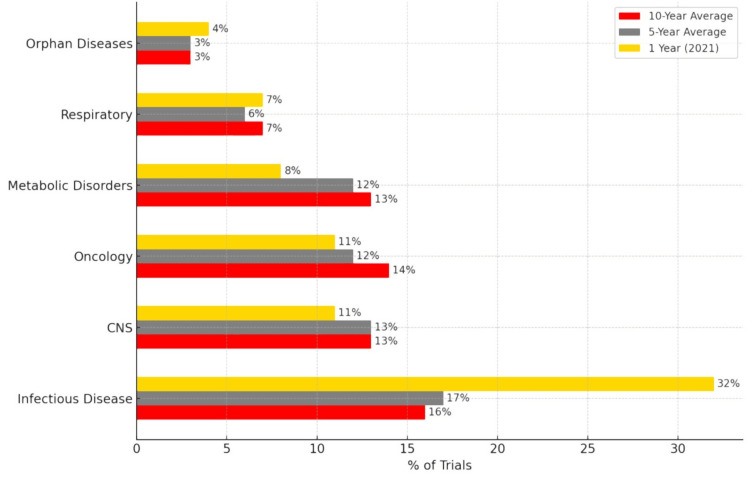
Site-wise distribution of clinical trials in India. The graph is reproduced with permission from PricewaterhouseCoopers (PwC) India report [80]. CNS: central nervous system.

This meticulous fine-tuning of the clinical trial regulatory structure has laid the groundwork for innovation and growth of the pharma sector. This is exemplified by a PricewaterhouseCoopers International Limited (PwC) report, which estimates the Indian clinical trials market to grow significantly, reaching $3.88 billion by 2030 [80]. India remains underrepresented in global clinical trials despite its large population and 8% of the worldwide cancer burden. It accounts for only 2% of global cancer trials. Indian participants make up only 2.9% of global clinical trial participants, in stark contrast to the 30% represented by the U.S. With 3.3% representation in oncology among pharmaceutical-sponsored breast cancer trials worldwide, breast cancer is the leading focus in India. To better understand the distribution of industry-sponsored clinical trials across different therapy areas in India over the past decade, please refer to the detailed data illustrated in Figure 12 below [80].

Despite these impediments, a hopeful light in The Clinical Trial Networks Initiative, launched by the National Biopharma Mission, aims to bolster India's capacity to conduct clinical trials in oncology, ophthalmology, rheumatology, and diabetology (CHOORD). This initiative spans five specialty networks, comprising 36 organizations (hospitals and research institutes) across 18 states. To address existing challenges, the National Biopharma Mission (NBM) will support the creation of clinical trial networks (CTNs) and enhance the nation's clinical trial capabilities. This initiative marks a promising step forward in advancing clinical research in India [81]. A comparable endeavor is the National Cancer Grid [82,83], which is an Indian network that links various organizations involved in cancer care and research. These organizations include major cancer treatment centers, research institutions, patient advocacy groups, and charitable organizations. It also aims to improve and standardize cancer care and research efforts across India. Butler [84] described the transition from bench to bedside as the "valley of death" in translational research, where more than two-thirds of compounds fail. The loss incurred is financial, scientific, and societal. Subbiah [85] has proposed modern, faster "go/no-go" decision trial designs as remedial measures in contemporary clinical research. The newer innovative trial designs, such as the umbrella study, basket study, platform study, and master observational trial, each represent a distinct trial design. They propose independent arms with control interventions, providing added flexibility. Table 4 presents a summary of these trial designs [85-87]. The I-SPY (Investigation of Serial Studies to Predict Your Therapeutic Response with Imaging and Molecular Analysis) breast cancer trial [88] and VE (Vemurafenib) basket study [89] have been well-received.

**Table 4:** A brief overview of the modern trial designs. The tabulated data are compiled from various prominent sources [85-87]. I-SPY: Investigation of Serial Studies to Predict Your Therapeutic Response with Imaging and Molecular Analysis; COVID-19: coronavirus disease 2019.

Design Type	Description	Example
Umbrella study	Evaluates multiple targeted therapies for the same disease entity, stratified by molecular alteration	I-SPY breast cancer trial
Basket trial	Tissue-agnostic studies where targeted therapy is evaluated on multiple disease types with the same molecular aberration	Vemurafenib basket study
Platform study	Multi-arm, multistage study designs comparing several intervention groups with a common control group, often perpetual with flexible design	Randomized evaluation of COVID-19 therapy; platform study
Master observation trial	Prospective, observational study design accepting patients independently of biomarker signature, collecting comprehensive data	Registry of oncology outcomes associated with testing and treatment

Moreover, with the increasing induction of AI-driven data analyses, the SPIRIT-AI extension for trial protocols [90] and the CONSORT-AI extension for publications [91] have been designed to ensure standardized and transparent reporting of randomized clinical trials involving AI. These guidelines mark the onset of a new era in clinical research modernization. As India undergoes significant metamorphosis in its clinical research arena, adopting these futuristic trial designs and standards can significantly enhance the quality and reliability of its clinical trials, driving better health outcomes.

Weaving the mosaic together

As we weave the intricate mosaic of India's healthcare transformation, we find ourselves at the convergence of history, innovation, and resilience. The country's prominent role in combating COVID-19 showcases its ability to harmonize various strengths into a unified, effective response. This experience, highlighted by swift vaccine development and the international recognition of digital tools such as CoWIN, demonstrates how a country can leverage its extensive resources and technological expertise to tackle extraordinary global health crises. India's robust pharmaceutical sector, known for its vast reach and high production capacity, is a vital cog in ensuring affordable and accessible healthcare worldwide. India's continued commitment to bridge the digital divide, reduce healthcare disparities, and expand medical service coverage using these transformative technologies underscores its dedication to creating a more equitable and advanced healthcare system for its diverse population. This commitment, coupled with initiatives in the pursuit of India-specific genomic data and the integration of precision medicine, represents a comprehensive approach to tailoring healthcare solutions that address the unique needs of the Indian populace, marking a significant step forward in the country's healthcare evolution.

The optimism for a brighter, healthier future remains undiminished in this tapestry of challenges, opportunities, timelines, and deadlines. The mosaic of India's healthcare system, with its vibrant colors of digital prowess, genomic research, and clinical excellence, continues to evolve. By weaving together these diverse elements, India is crafting a robust healthcare framework for its population and setting a precedent for global health leadership.

## Conclusions

India finds itself at a transformative crossroads in its healthcare evolution, with cancer care emerging as a critical frontier for innovation and progress. The country's technological trailblazing demonstrated through its digital health initiatives, such as CoWIN and the ABDM, positions it uniquely to leverage advanced technologies in healthcare. The potential of India's robust IT infrastructure to revolutionize healthcare innovation is a cause for optimism.

India's pharmaceutical sector, renowned globally as the "pharmacy of the world," is a testament to the nation's formidable prowess in drug innovation, large-scale manufacturing, and global distribution networks. This powerhouse industry underscores India's dominance in the pharmaceutical landscape and highlights its pivotal role in ensuring global access to life-saving medications. Opportunities abound in genomic research, with initiatives such as GenomeIndia and the Indian Cancer Genome Atlas Foundation paving the way for personalized medicine. The clinical trial landscape is evolving, with regulatory reforms making India an attractive destination for global studies. Adopting innovative trial designs and AI-driven analyses could further enhance this sector. However, challenges persist. Despite its large population and significant cancer burden, India remains underrepresented in global clinical trials. Financial toxicity in cancer care remains a major concern, highlighting the need for affordable treatment options. The country's genomic databases are still in their infancy, accounting for a mere 0.2% of global genetic data. India's strengths lie in its IT infrastructure, robust pharmaceutical sector, and large, diverse population. These assets can be leveraged to develop India-specific cancer care solutions. While India faces areas for growth in global genomic representation and clinical trial participation, these present unique opportunities for advancement. The increasing cancer prevalence and potential for healthcare inequities underscore the urgency for innovative solutions. Yet, these challenges herald a promising future. By leveraging its digital prowess, accelerating genomic research, and strategically enhancing its clinical trial landscape, India stands poised to emerge as a pioneer PPCM. Crucial to this transformation is establishing an India-specific biobank, drawing inspiration from successful initiatives such as the Qatar Biobank. Such a resource would catalyze research tailored to India's diverse population and address the current underrepresentation in global genomic databases. This holistic approach could revolutionize cancer care within India and beyond, potentially serving as a blueprint for other developing nations in the Global South. By turning challenges into opportunities, India is on the cusp of a healthcare transformation that could redefine the landscape of precision oncology on a global scale.

## Appendices

Definitions

Knowledge translation: the process of turning research (cancer research in this context) findings into practical actions that improve patient care and outcomes in Indian hospitals and communities. It is about ensuring that the latest cancer treatments and prevention strategies actually reach and benefit patients across India.

Genetic medicine (GM): medicine based on genetics (including "omics") profiling of individuals (subjects) in disease management, prevention, and counseling.

Precision medicine (PM): medicine based on a multidimensional assessment of an individual/patient/subject, leading to individualized/customized disease management/prevention and counseling. PM is more than GM. For example, it includes big data, telemedicine, and electronic health records.

Precision population medicine (PPM): an interdisciplinary and multi-specialty approach to improving individual and population health. It encompasses population/special population health input and integrates various aspects of healthcare to provide targeted, personalized interventions. It includes all the items included in PM plus public/population health science applications.

Precision population cancer medicine (PPCM): an innovative take on precision population medicine (PPM), as it extends this concept specifically to cancer patients to enhance cancer care outcomes.

Financial toxicity: in cancer care, it refers to the severe financial strain experienced by patients and their families due to high treatment costs and prolonged treatment, often resulting in debt, bankruptcy, or the need to forgo necessary care.

The current state of cancer care in India

Outcome metrics: the survival outcomes are generally inferior compared to those of developed countries, with significant urban-rural disparities.

Strengths: a growing network of specialized cancer centers, digital and technological infrastructure, pharmaceutical industry, and increasing awareness of early detection.

Weaknesses: limited access to advanced treatments in most areas and high out-of-pocket expenses for patients.

Opportunities: telemedicine and digital health have the potential to improve rural access, along with increased government focus on expanding cancer care infrastructure.

Threats: increasing cancer incidence due to lifestyle changes, shortage of oncology-specific medical equipment and facilities, and treatment abandonment due to financial constraints.

Summary of current Indian programs that can help improve PPCM in India

CoWIN (winning over COVID-19): the application has been developed as a comprehensive cloud-based IT solution for planning, implementation, and monitoring of COVID-19 vaccination in India.

Ayushman Bharat Digital Mission (ABDM): launched in 2021, ABDM aims to connect the digital health solutions of hospitals across the country to ensure an integrated digital health infrastructure in India.

GenomeIndia: a visionary national project funded by the Department of Biotechnology, Government of India. It was launched in January 2020. Its goal is to sequence 10,000 genomes from healthy Indian individuals across the country. The primary aim is to create a precious national resource for India's public health. The objective is to revolutionize healthcare, empowering basic researchers and clinicians, leading to transformative precision interventions.

Indian Cancer Genome Atlas (ICGA): initiated in 2020-2021 by forming a national consortium of government agencies, cancer hospitals, academic institutions, and private sector partners in India. The ICGA is a long-term collaborative effort between cancer scientists, basic researchers, onco-clinicians, data scientists, and technology providers to facilitate precision medicine and improve translational cancer research in India.
